# Effects of Climate Change on Range Forage Production in the San Francisco Bay Area

**DOI:** 10.1371/journal.pone.0057723

**Published:** 2013-03-05

**Authors:** Rebecca Chaplin-Kramer, Melvin R. George

**Affiliations:** 1 Natural Capital Project, Stanford University, Stanford, California, United States of America; 2 Plant Sciences Department, University of California Davis, Davis, California, United States of America; DOE Pacific Northwest National Laboratory, United States of America

## Abstract

The San Francisco Bay Area in California, USA is a highly heterogeneous region in climate, topography, and habitats, as well as in its political and economic interests. Successful conservation strategies must consider various current and future competing demands for the land, and should pay special attention to livestock grazing, the dominant non-urban land-use. The main objective of this study was to predict changes in rangeland forage production in response to changes in temperature and precipitation projected by downscaled output from global climate models. Daily temperature and precipitation data generated by four climate models were used as input variables for an existing rangeland forage production model (linear regression) for California’s annual rangelands and projected on 244 12 km x 12 km grid cells for eight Bay Area counties. Climate model projections suggest that forage production in Bay Area rangelands may be enhanced by future conditions in most years, at least in terms of peak standing crop. However, the timing of production is as important as its peak, and altered precipitation patterns could mean delayed germination, resulting in shorter growing seasons and longer periods of inadequate forage quality. An increase in the frequency of extremely dry years also increases the uncertainty of forage availability. These shifts in forage production will affect the economic viability and conservation strategies for rangelands in the San Francisco Bay Area.

## Introduction

California’s San Francisco Bay Area is a mosaic of urban and natural lands, and tension exists between the two with scenic beauty attracting development that threatens these prized open spaces [1]. About half of the area of the eight San Francisco Bay counties is classified as rangeland, and these areas account for most of the region’s open space. The private ranches on Bay Area rangelands provide a livelihood and a way of life that helps to limit urban sprawl [2]. With a growing population placing pressure on these areas for development and a changing climate posing new threats to rangeland ecosystems, understanding the degree to which their value as working landscapes will be maintained in the future is important to their conservation.

**Figure 1:**
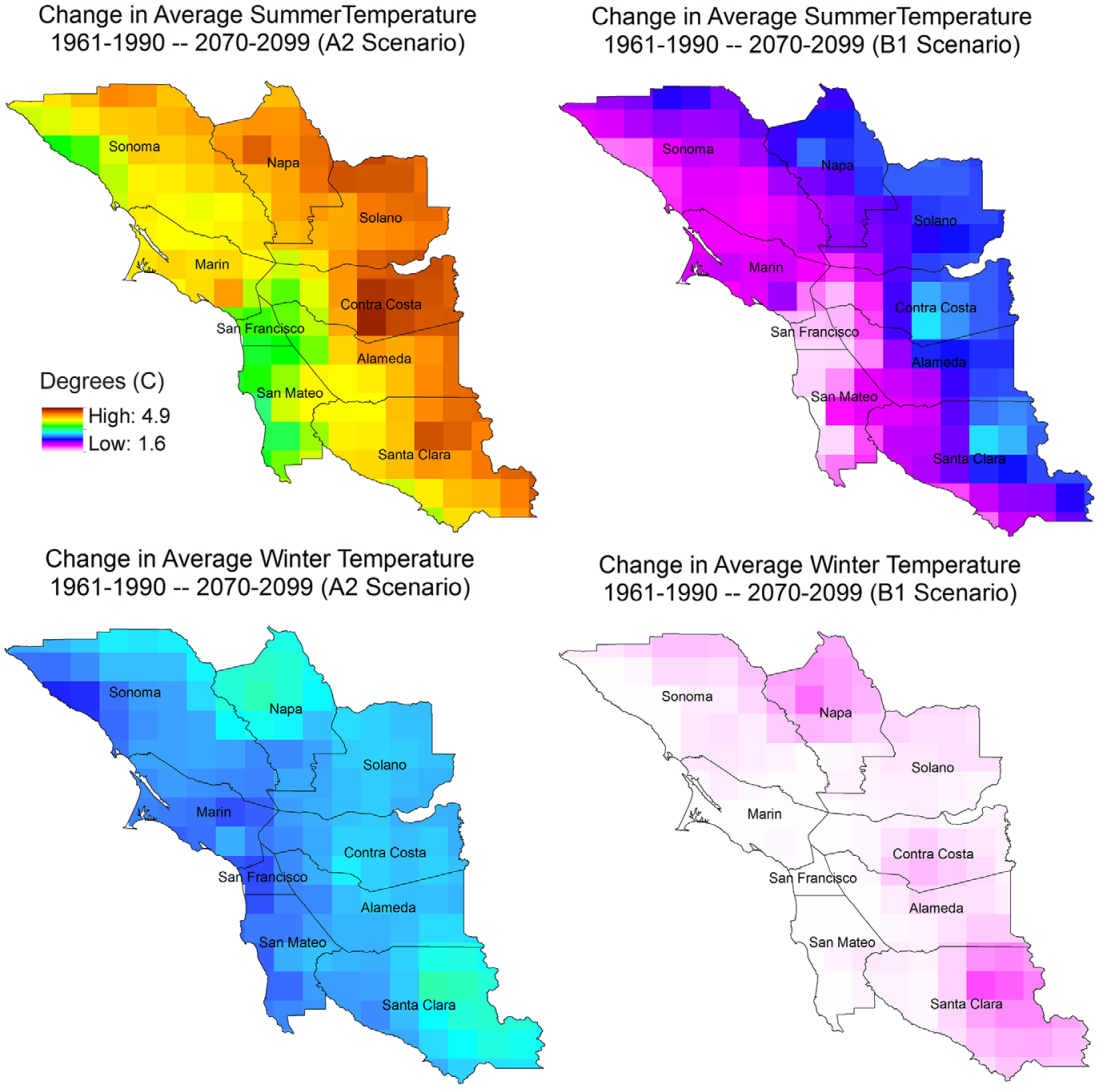
Historical (1961–1990) and projected (2070–2099) average temperatures for summer (June, July, August) and winter (January, February) months in the Bay Area. Temperatures reflect means of four global climate models (downscaled output from CNRM CM3, GFDL CM2.1, NCAR CCSM3.0 and NCAR PCM1).

Over the next century California temperatures are projected to rise between 1.7°and 3.0°C for a lower emissions scenario, and 4.4° to 5.8°C for a higher emissions scenario [3]. Downscaled results from global climate models for the San Francisco Bay Area show a lower rise in temperatures, from 1.5° to 3.0°C by 2100 for the lower emissions scenario and 2.5° to 4.4°C for the higher emissions scenario, though considerable variation exists within the region (Fig. 1). Changes in precipitation are more uncertain, with a high degree of variability between different climate models, and even from year to year within the same model, suggesting that the region will remain vulnerable to drought. Overall, the majority of simulations indicate that total annual precipitation will decline, mostly in the spring months, while winter precipitation will remain relatively stable [4].

The full extent of climate change impacts on rangeland forage production in California and the Bay Area in particular is uncertain. One study [5] modeled the impact of forecasted changes in precipitation patterns on California rangeland production and concluded that areas of the state suitable for cattle grazing would shift, as some areas become wetter and others become drier, depending on the climate model. Statewide, they predicted range forage production would decline between 14 and 58 percent, corresponding to a reduction in annual profits from cattle ranching of between $22 million and $92 million by 2070. Despite this statewide trend, their results for the Bay Area suggested the impacts would be more positive, with production increases projected for Santa Clara, Alameda, Contra Costa, Solano, and Napa Counties. Marin and Sonoma Counties were not included in the model. However, this precipitation-based model did not incorporate rangeland response to warming, and the authors acknowledged that their model may have overestimated the effects of precipitation. While precipitation has been shown to be an important variable for predicting annual rangeland productivity, temperature within the growing season is also important [6]. Precipitation, temperature and forage production data collected since 1935 at the San Joaquin Experimental Range in Madera County have shown that near average production can occur in low rainfall years if precipitation is well distributed and low annual production can occur in wet years if precipitation is poorly distributed or if temperatures are below normal, as is often associated with wet weather [7]. Thus, integrating changes in both temperature and precipitation could improve forecasts of rangeland forage production.

The combined effects of seasonal temperature and precipitation patterns will influence not only productivity, but also growing season length and plant phenology in rangelands [8]. Warming has been shown to increase soil water content by accelerating plant senescence [9], which may interact with changes in precipitation to further affect water availability in rangelands. In fact, grassland ecophysiology may be less responsive to changes in total quantity of rainfall than to shifts in seasonal patterns of rainfall [10]. Early-season precipitation alone explained 49 percent of the variability in shoot-growth at the University of California Hopland Research and Extension Center (UC HREC), just north of the Bay Area [11], although additional data reduced this explanatory power to 34 percent [6]. Late-season precipitation also has a pronounced impact on Bay Area and North Coast grassland production, shown by increased shoot growth resulting from experimental water additions in the late spring [12], [13].

Despite these known links between the effects of warming and precipitation in grasslands, no models incorporating the impact of warming on California rangeland production yet exist. Increases in spring plant production and an extension of the growing season has been predicted for the Great Plains [14]. However, these authors warned that increases in variance may be more important than the mean effect, because uncertainty in predicting plant growth results in suboptimal stocking decisions. They suggested that the increased variance found in their simulations would require carrying capacities to decrease from about 6.5 to 9.0 ha per animal, in order to maintain a 90 percent confidence of not overstocking. Further, more intense management would increase operating costs, and therefore may negate any benefits in forage production. In contrast to the Great Plains, where the growing season begins in the spring months following winter dormancy, the Bay Area rangeland growing season begins with the first fall rains and ends with soil moisture depletion in the spring months. Climate change in the Bay Area may be more comparable to that found in the Mediterranean climate of southern Australia. For this region, lower pasture production has been projected for future climates with lower precipitation and higher temperatures [15].

Incorporating the effects of warming into models of rangeland production in California, and the Bay Area in particular, is an important step in understanding how climate change will affect range livestock production in this region. The economic viability of rangelands is essential to maintaining the natural aesthetic that contributes to the quality of life of the residents of this unique urban-natural interface. This paper reports the projected changes in forage production in response to simulated future temperature and precipitation in the San Francisco Bay Area to better understand how climate change will impact these working landscapes so important to local conservation.

## Methods

### Study Area

The geographic diversity of the San Francisco Bay Area (hills, mountains, and large water bodies) produces a wide variety of microclimates. Coastal areas are generally characterized by relatively small temperature variations during the year, with cool, foggy summers and mild, rainy winters. Inland areas, especially those separated from the ocean by hills or mountains, have hotter summers and colder overnight temperatures during the winter. The rangelands in the North Bay (with its northwestern most point at 38.9375N, 123.6875W; encompassing Sonoma, Marin, Napa, and Solano Counties) are characterized by higher rainfall, a longer rainy season and cooler temperatures than those in the South Bay (southeastern most point at 36.9375N, 121.1875W; encompassing Santa Clara, Alameda, and Contra Costa Counties). San Jose, at the south end of the Bay averages fewer than 380 mm of rain annually, while Napa, in the North Bay area, can exceed 750 mm. Because range forage production is strongly influenced by temperature and precipitation, there are significant differences in growing season length and productive potential between the North and South Bay areas.

### Climate Models

Climate data were acquired from Cayan et al., downscaled from global climate models using a Bias Corrected Constructed Analogues (BCCA) technique to produce two climate scenarios: the lower-emissions B1 scenario and the higher-emissions A2 scenario [4]. Four climate models produce daily temperature and precipitation projections: Centre National Recherché Meteorologique (CNRM) CM3, Geophysical Fluid Dynamics Laboratory (GFDL) CM2.1, National Center for Atmospheric Research (NCAR) CCSM3.0, and NCAR PCM1. Each climate model was back-cast to simulate historical climate conditions (1961–1990) and represented historical climate data with accuracy [4].

### Forage Production Model

Throughout California’s 14.5 million acres of annual rangelands, which include the Bay Area grasslands and oak woodlands of this study, temperature is the main constraint to productivity during the growing season. Therefore, precipitation and evapotranspiration drove a simple model to determine growing season length, and temperature and growing season length drove the model for annual forage production.

Daily climate data from the four climate models were input variables for a forage production model reported by Californian researchers [16] who found that growing degree days accounted for 75 to 95 percent of the variation in growing season production (Table 1). This degree-day forage production model was run for all eight model/scenario combinations (described in Climate Models, above), for each of the 244 12 km x 12 km grid cells that comprise the Bay Area region. The mean of the output from the four climate models was taken for the A2 and B1 scenarios, and compared to output for a simulated historical period (1961–1990). All calculations and simulations were produced in the R software package [17].

**Table 1:** Relationship between forage production (y, kg ha^−1^) and accumulated degree days (x) from 10 sites in four annual rangeland counties [16].

Sample Area	County	Latitude (N)	Longitude (W)	Regression Equation	R^2^
1	Yuba	39.330361	121.3476	y = −120+5.2x	0.95
2	Butte	39.384564	121.59456	y = 14+4.4x	0.91
3	Madera	37.088669	119.73461	y = −90+3.8x	0.85
4	Madera	37.089609	119.713978	y = −141+3.1x	0.82
5	Madera	37.089811	119.73698	y = −54+3.9x	0.77
6	Madera	37.095235	119.736424	y = −280+4.9x	0.88
7	Mendocino	38.997164	123.092492	y = 77+2.2x	0.74
8	Mendocino	38.986653	123.08472	y = 138+2.8x	0.76
9	Mendocino	39.006414	123.085347	y = 96+4.1x	0.91
10	Mendocino	39.003462	123.077236	y = 82+2.7x	0.74

Modeling the bounds of the growing season was necessary to convert a regression model based on field data into a predictive model to simulate forage production under future climate scenarios. A germinating rain that exceeds 25 mm within one week marks the start of the growing season [16]. There is no similarly well-established climatic phenomenon marking the end of the growing season; in field studies it is determined empirically, by measuring biomass until annual grasses are between the soft and hard dough stage of seed maturity [16]. Therefore, the end of the growing season in this study was simulated using a simple water balance model that was trained using CIMIS weather data for precipitation and evapotranspiration from the University of California Sierra Foothill Research and Extension Center (UC SFREC), 17 miles northeast of Marysville, California. Calculating the point at which cumulative evapotranspiration exceeded cumulative precipitation over a moving window of 60 days best predicted the peak forage date. This simple model generally came within two weeks of actual peak forage date measured at the UC SFREC, rarely extending beyond the end of May. For each year, germination and season end dates were computed according to these methods, and set the seasonal bounds within which forage production was modeled, capturing inter-annual variability in season length.

To simulate forage production, degree-days were first calculated from model-generated minimum and maximum daily temperatures above a base temperature of 5°C using the sine function method [16], [18]. Accumulated degree-days (ADD), the sum of all previous degree-days from a given date, were calculated at monthly intervals from germination until the end of the growing season. Monthly standing biomass or total forage production was estimated from ADD using the regression equations from several annual rangeland sites in Table 1 [16]. Absolute forage production varied depending on the chosen equation, but as the relationship is linear, relative measures such as the change in forage production over time were very consistent, differing by only 2 to 3 percent. For this reason, future values for peak forage production are presented in terms of change from historic values.

Growth curves were constructed using the monthly forage production estimates, averaged over the window of historic (1961–1999) and future (2070–2099) time periods for both A2 and B1 emissions scenarios, and then averaged over all rangeland area in a county. These growth curves help to determine which parts of the season have the greatest differences between historical and future conditions, and thus hint at the mechanisms behind the difference. Differences in the first month may indicate that germination date is an important factor. Steeper slopes throughout the middle of the curve would point to the role played by warmer winter and/or spring temperatures. Differences in the slope leading up to the final time step could be at least partially explained by differences in season end date and the length of time for degree days to accumulate in that final period. Months are taken as calendar months, such that if germination occurred on September 23, the month of September would only have one week of forage production. Likewise, if the end of season date is calculated as June 2, the month of June would only have two days of additional forage production added to the overall total.

All model calculations were repeated for every year of simulated climate data from 1961 to 2099, and the data were summarized by taking the means in four windows of time: historic (1961–1999), early century (2005–2034), mid-century (2035–2064), and late century (2070–2099). Changes in peak production and season length from historic to future conditions are presented on maps of rangeland biomes (savannah, grasslands and shrublands) selected from the Existing Vegetation Types layer of the national LANDFIRE dataset [19] and overlaid on the model outputs.

## Results

### Forage Production

**Figure 2:**
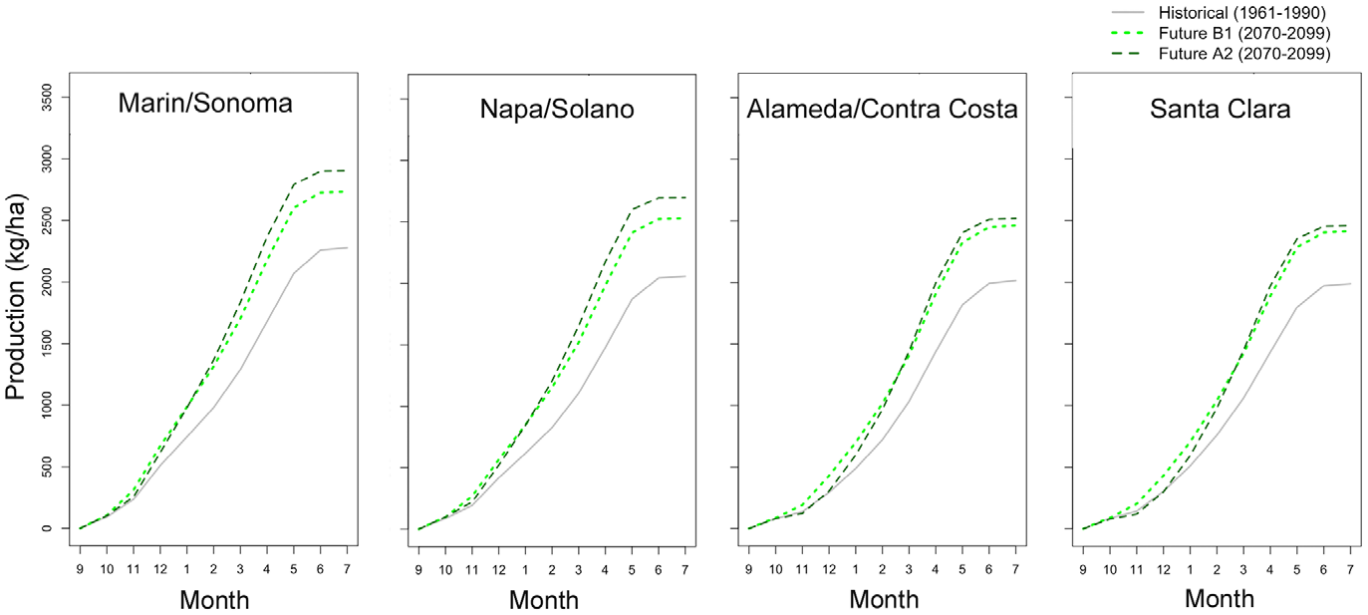
Seasonal growth curves for forage production in different regions under historical (1961–1990) and future (2070–2099, for A2 and B1 emissions scenarios) climate conditions. Growth curves represent the accumulated forage produced on a daily time-step, summarized at monthly intervals as the amount of total forage produced over the season by that date. Each line shows the mean production for all years within each 30-year period and for all cells within each county. Only cells containing rangelands were used (see Figure 3).

**Figure 3:**
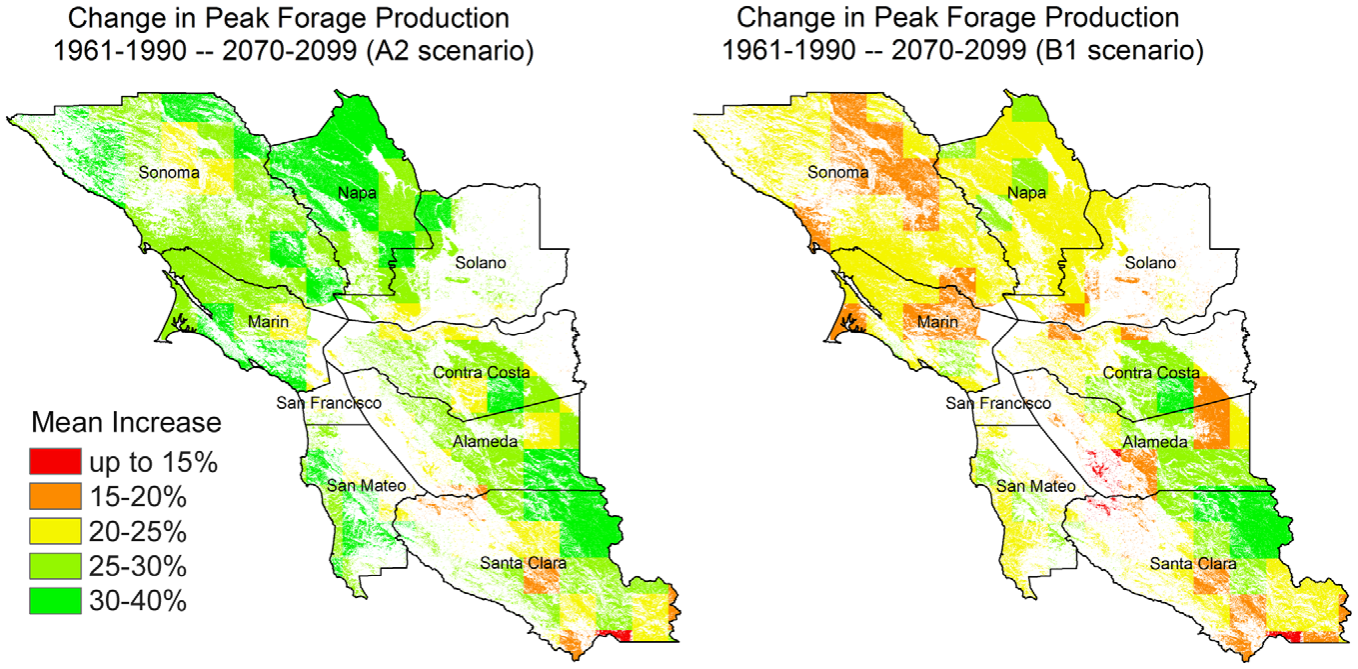
Change (%) in peak forage production by late-century (2070–2099), relative to historical conditions (1961–1990), shown for current rangelands (grassland, savannah, and shrubland) in the Bay Area.

Forage production by the end of the century (2070–2099) increases in each month of the growing season relative to simulated historical forage production (1961–1990), resulting in increased total forage production under future climate conditions throughout the San Francisco Bay Area (Fig. 2). There is little difference between projections for historic vs. early-century (2005–2034) and mid-century (2035–2064) forage production for either scenario. Projected mid-century peak forage production increased only 10 to 13 percent from the historical (1961–1990) conditions for the higher-emissions A2 scenario (Table 2). However, average production for each of the eight Bay Area Counties is projected to increase 24 to 31 percent by the late century (2070–2099) for the A2 scenario, with increases of up to 40 percent in much of Northern Napa and Sonoma Counties (Fig. 3). Projected increases for these counties in the B1 scenario are slightly higher by mid-century and more modest at the end of the century. However, late-century northeastern Santa Clara County shows an increase above 30 percent for both emission scenarios.

**Table 2:** Change in peak forage production and season length compared to historic (1961–1990) conditions (mean +/− standard error).

	Marin-Sonoma		Napa-Solano		Alameda-Contra Costa		Santa Clara	
Time Period	Mean	+/−	Mean	+/−	Mean	+/−	Mean	+/−
	Change in Forage Production (%): A2 Model							
2005–2034	10.1	2.2	10.4	2.5	13.1	2.8	11.8	2.5
2035–2064	11.9	2.5	13.1	2.9	13.5	3.0	13.0	3.0
2070–2099	27.5	2.8	31.1	3.0	24.9	3.1	23.6	2.9
	Change in Forage Production (%): B1 Model							
2005–2034	9.4	2.9	10.1	3.1	9.5	2.9	10.8	3.1
2035–2064	16.2	2.9	19.2	3.2	17.8	3.5	17.1	3.6
2070–2099	20.0	2.6	23.0	2.8	22.1	3.1	21.5	3.1
	Reference Historic Season Length (Total Days)							
1961–1990	181.1	2.5	171.8	2.5	152.8	2.78	155.3	2.9
2005–2034	−1.7	2.3	−2.4	2.5	0.3	2.9	−1.2	2.8
2035–2064	−9.7	2.4	−9.2	2.5	−9.6	3.0	−10.9	3.0
2070–2099	−15.5	2.6	−13.7	2.6	−16.3	2.8	−19.4	2.5
	Change in Season Length (Days): A2 Model							
2005–2034	−2.9	3.1	−3.1	3.2	−3.4	3.6	−2.5	3.8
2035–2064	−3.3	3.0	−1.9	3.1	−3.6	3.4	−4.6	3.3
2070–2099	−5.1	2.8	−4.1	2.7	−3.3	3.4	−6.2	3.4
	Change in Season Length (%): A2 Model							
2005–2034	−0.9	1.3	−1.4	1.5	0.2	1.9	−0.7	1.8
2035–2064	−5.4	1.3	−5.3	1.5	−6.3	2.0	−7.0	1.9
2070–2099	−8.6	1.4	−8.0	1.5	−10.7	1.8	−12.5	1.6
	Change in Season Length (Days): B1 Model							
2005–2034	−1.6	1.7	−1.8	1.8	−2.2	2.4	−1.6	2.4
2035–2064	−1.8	1.6	−1.1	1.8	−2.3	2.2	−3.0	2.1
2070–2099	−2.8	1.5	−2.3	1.6	−2.1	2.2	−4.0	2.2

### Season Length

**Figure 4:**
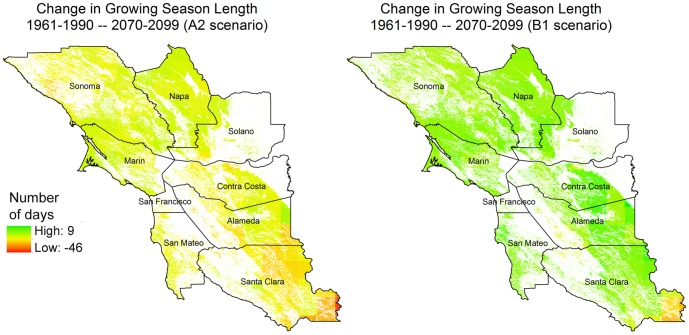
Change in rangeland season length by end-century (2070–2099), relative to historical conditions (1961–1990) for current rangelands in the Bay Area.

The models also predicted changes in growing season length, due to changes in the simulated timing of germination (precipitation exceeding 25 mm in one week) in the fall and soil moisture depletion (evapotranspiration exceeding precipitation over a 60 day period) in the spring. The length of the growing season is projected to markedly decrease under the A2 scenario; two-week shorter seasons can be expected by late-century for much of the Bay Area (Table 2), with parts of Santa Clara showing seasons shrinking by more than three-weeks (Fig. 4). The B1 scenario shows more modest decreases in season length of a few days to a week. With decreasing rainfall, future forage season lengths in eastern Santa Clara and Alameda Counties could drop to as low as 100 days in length, a full 50 days shorter than the shortest season found in Marin or Sonoma Counties.

### Inter-annual Variability

The standard errors reported in Table 2 indicate that inter-annual variability in peak forage production and season length is fairly low in the Bay Area, under both historic and future conditions. These standard errors range from 1 to 3 percent of the mean for either of these variables in any county. On the other hand, the maximums and minimums for each 30-year time period range from 38 percent below to 53 percent above the mean forage production and 29 percent above to 46 percent below mean season length (Table 3). More information can be gleaned by considering the variability as the spread of the distribution, the number of data points falling outside a range of 2 standard errors around the mean (Table 4). By this definition, inter-annual variability in peak production declines fairly dramatically throughout most of the Bay Area for both scenarios, due mainly to the number of years that production is above average in the North Bay, while Alameda and Contra Costa shows declines in variability around both sides of the mean for the B1 scenario and in the number of years production is below average in the A2 scenario. Overall inter-annual variability in peak production does not change in Santa Clara, but is skewed more negatively in future climate conditions under both scenarios (more years in which peak production falls below 2 standard errors below the mean occur in the future than historic conditions). Inter-annual variability in season length remains the same or even declines for most counties for the B1 scenario. In the A2 scenario variability increases slightly, with more years falling farther below average season length than historically. The variability is more centered around the mean in all future scenarios than in historical climate conditions (which were skewed slightly positively; more longer than average seasons than shorter than average seasons).

**Table 3:** Maximum and minimum forage production and season length, expressed as percent above and below the mean for the historic period (1961–1990), three projected 30-year periods and all periods.

	Marin-Sonoma		Napa-Solano		Alameda-Contra Costa		Santa Clara	
Time Period	Max	Min	Max	Min	Max	Min	Max	Min
	Production A2 Model							
All periods	45	32	50	34	45	28	49	35
1961–1990	23	24	30	25	34	19	33	27
2005–2034	19	24	25	24	26	26	23	17
2035–2064	24	18	30	19	36	23	41	22
2070–2099	28	24	29	26	31	29	34	23
	Production B1 Model							
All periods	35	31	39	33	53	38	46	37
1961–1990	23	24	30	25	33	19	33	27
2005–2034	32	22	35	22	30	34	35	37
2035–2064	23	34	27	31	28	30	26	34
2070–2099	25	22	27	23	40	24	34	22
	Season Length A2 Model							
All periods	24	23	24	24	26	38	26	32
1961–1990	13	18	17	20	21	17	21	25
2005–2034	15	13	16	11	22	15	20	15
2035–2064	15	18	17	15	25	19	29	17
2070–2099	22	17	24	17	26	32	29	26
	Season Length B1 Model							
All periods	22	26	21	25	28	46	24	39
1961–1990	13	18	17	20	20	17	21	25
2005–2034	18	16	20	23	21	36	23	39
2035–2064	15	25	14	21	24	22	21	22
2070–2099	16	15	15	14	28	30	24	29

**Table 4:** Number of outlier years (>2 standard errors above or below the mean) per time period.

	Marin-Sonoma	Napa-Solano	Alameda-Contra Costa	Santa Clara
	Peak Forage Production			
Below historic mean	12	11	13	10
Above historic mean	11	10	10	10
Below mean for 2070–2099 (A2)	10	11	10	11
Above mean for 2070–2099 (A2)	7	8	10	9
Below mean for 2070–2099 (B1)	11	10	10	13
Above mean for 2070–2099 (B1)	8	8	7	8
	**Season Length**			
Below historic mean	9	10	10	7
Above historic mean	12	12	10	11
Below mean for 2070–2099 (A2)	10	12	11	10
Above mean for 2070–2099 (A2)	10	11	10	9
Below mean for 2070–2099 (B1)	11	11	7	7
Above mean for 2070–2099 (B1)	11	10	8	8

### Drought Years

The climate models predict that some regions in the Bay Area would see some years with no germinating rains and therefore no growing season. Historically this would generally occur in any given location in the South Bay once over a 30 year period. While comparisons of model projections revealed that there is a high degree of variation among the four climate models, the mean shows a lower frequency of non-germination years in the southern counties for the B1 scenario compared to historic conditions, and a higher frequency for the A2 scenario. Specific outcomes supported by all models and for both scenarios are that the North Bay is almost entirely unaffected in all time periods and southeastern Santa Clara County experiences more extreme dry years under future conditions.

## Discussion

### Forage Production

Our model supports the results of earlier efforts incorporating only precipitation into a model for forage production in the Bay Area [5], though the increases in forage production seen here were in spite of, not because of, a shift in precipitation patterns. The agreement between these two models stands in contrast to the projections of a similar Mediterranean climate in Southern Australia [15], where lower forage production was expected under warmer and drier climate. This difference may be due to the fact that precipitation is not as limiting in the Bay Area system as winter temperatures. Projected warming for A2 and B1 scenarios result in higher standing crop throughout the growing season and at the end of the growing season for the 2070–2099 period compared to the historical period (Fig. 2).

**Figure 5:**
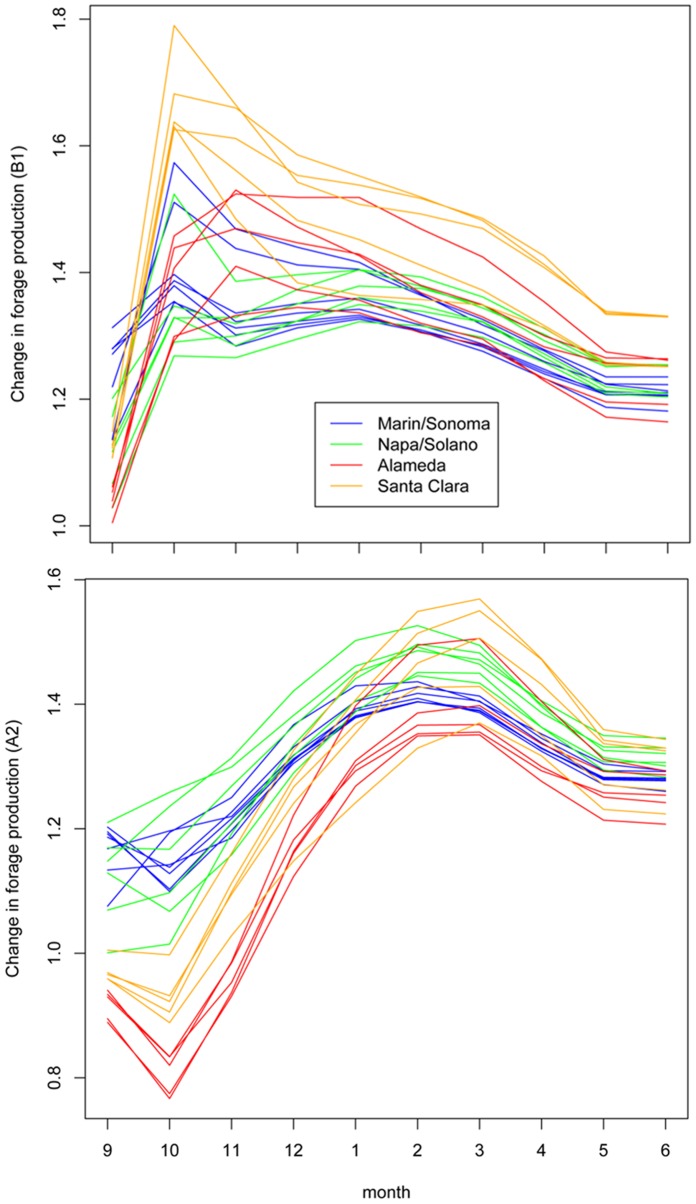
Change (%) in forage production from historical (1961–1990) to future (2070–2099) climate scenarios. Multiple lines of the same color represent different 12 x 12 km grid cells of rangeland in that area.

Comparisons of historical monthly production to projected production for the two scenarios reveal that production increases in the A2 scenario reach a maximum (greatest difference from historical conditions) in most parts of the Bay Area by the beginning of March or April (Fig. 5). In the B1 scenario, the maximum change from historical conditions occurs much earlier, by the end of October or November, but the magnitude of the maximum differences between historic and future production are much more variable than in the A2 scenario (25 to 80 percent increases for B1, 30 to 55 percent for A2). This difference between the two scenarios is likely at least partially due to earlier onset of germinating rains in the B1 scenario, as discussed below in Season Length. In the early season, when total forage production is very low, even a small absolute change in production made by a few days or a week of extra production time can make a large difference proportionally.

### Season Length

In this model, season length changes with the timing of germination and plant senescence; delayed onset of germinating rains and/or earlier depletion of soil moisture will result in shorter growing seasons. Because forage quality is greatest during the growing season [20], periods of adequate forage quality for animal production will be shortened under future climate conditions, despite increases in forage production. The main differences between scenarios and across different regions in this study are due to the timing of germination. In the A2 scenario, the growing seasons in Santa Clara and Alameda are delayed by a week to 12 days compared to historical conditions, whereas the season in the northern Bay Area starts only slightly (2–3 days) later than historically. This intensifies the historical differences between germination dates of the North and South Bay. In contrast, earlier rains mean earlier germination (2–3 days on average, up to a week) throughout much of the Bay Area for the B1 scenario, which almost compensates for the earlier end in the B1 scenario, such that the impact of climate change on season length is more subtle than for the A2 scenario. This earlier start to the growing season also contributes to greater production in the late-century B1 scenario compared to the A2 scenario during the first few months of the season (Fig. 2).

These changes in season length and timing could have major implications for the range livestock industry in the San Francisco Bay Area. For example, delaying the start of the growing season and associated improvement in forage quality could impact the traditional fall calving season, and earlier onset of the dry season could require early weaning of calves in cow calf operations that dominate Bay Area livestock production. If the timing of these key events changes, then breeding and marketing dates may also shift by a few days or weeks. With later germination and earlier end to the growing season, managers will seek to place stock on summer pasture, including public lands, sooner and keep them there longer.

### Inter-annual Variability and Drought

Inter-annual variation appears to decline with climate change throughout much of the Bay Area. This suggests that the uncertainty in stocking decisions that were cited as a major concern for climate change in the Great Plains [14] will be less of an issue here, and ranchers will likely not have to change their management response from what they do now to respond to low production years and good production years (see Conclusions). However, this low variability masks outlier years; the minimums of 38% below mean forage production do not include the years in which no forage is produced, due to drought. In fact, the occurrence of these “skipped” forage seasons can be considered an extreme case of inter-annual variability. While there exists a high degree of uncertainty over the Bay Area as a whole, extreme events in future precipitation forecast by the four different climate models are likely to increase in regions already most vulnerable to such events. Droughts so severe that forage is not produced during the growing season can be expected to increase in frequency in parts of the South Bay, which will have serious consequences for stocking decisions and the overall reliability of forage in that area.

### Model Limitations and Other Climate Impacts

This simple forage production model was developed from data taken across rangelands that represent much of the Bay Area; UC HREC is ten miles north of interior Sonoma County (east of the Coast Range), and the other two research stations (San Joaquin Experimental Range in Madera County and UC SFREC in Yuba County) are inland sites that are more similar to the eastern portions of the Bay Area counties. However, the coastal regions may not be accurately represented. The curves projected for the historical period in the coastal counties of Marin and Sonoma are steeper than for the inland counties of Alameda and Santa Clara and than those from earlier empirical studies [8], especially during winter months. This difference may be explained by the generally warmer winter temperatures forecast for Marin and Sonoma County rangelands than for Alameda and Santa Clara County rangelands. Therefore, while more coastal data would improve the accuracy of model, most of the coastal effects on productivity are likely temperature effects that should be adequately modeled by accumulated degree days.

One major simplification of the processes involved with forage production in this model is that precipitation is included only to define the bounds of the growing season [16]. Precipitation effects within the growing season are not considered in the model, and it therefore does not respond to midwinter droughts, which may substantially reduce forage production in some years. At most annual rangeland locations in the Bay Area, moisture is seldom limiting during the growing season [7]. The degree day model used in this study was developed using production and weather data from areas that vary quite dramatically in their precipitation regimes, with annual rainfall ranging from 13 to 53 inches [16] Modeled annual precipitation for Marin, Sonoma, Napa, and Solano Counties did not fall outside of this range in any year. Alameda, Contra Costa and Santa Clara Counties did show some years that fell below 13 inches (330 mm), but these generally accounted for <15% of the total years. The number of years falling below that range did not increase from historical to future conditions in the B1 scenario, which means the model can be applied with confidence to all regions of the Bay Area for this scenario. The number of years precipitation in the A2 scenario fell outside that range increased in Alameda/Contra Costa Counties from 1961–1990 to 2070–2099. Results should therefore be interpreted more cautiously for these counties in the A2 scenario.

Finally, the model leaves out a number of important processes determining forage production that may be significantly altered under future conditions. Elevated atmospheric carbon-dioxide could have fertilization effects that increase the quantity of forage, while simultaneously reducing the quality by diluting the protein content [21]. Future climate could alter evapotranspiration rates, resulting in decreased soil moisture and increased water stress beyond the effects of precipitation [22], further reducing season length and potentially increasing drought frequency. Potential shifts in vegetation states resulting from projected changes in temperature and precipitation can also impact forage production at the landscape level, through shrubland expansion into existing grasslands and long-term conversion of oak woodlands to grasslands [23]. Finally, animal metabolic performance, grazing behavior and availability of stock water can all be expected to change with climate [24], [25], [26], and while not modeled here, their general decline with temperature will impact the overall viability of the livestock industry in the Bay Area.

Incorporating these additional factors into a model of forage production would result in a more sensitive and nuanced forecast of this important ecosystem service under climate change conditions. Our goal in this research was to apply to future climate scenarios a very simple, empirical model that despite its simplicity explains 75% of the variation in forage production under current conditions in the Bay Area. Future research should compare a simple model such as that presented here with more complex approaches, to determine how well main effects are characterized by the most basic processes. Simple models can be useful for supporting land use decisions in areas where data are limited and/or more advanced processes are poorly understood.

### Conclusions

Climate change has the potential to impact the quantity and reliability of forage production, forage quality, thermal stress on livestock, water demands for both animal needs and growing forage, and large-scale rangeland vegetation patterns. This study projects increases in forage production within the growing season counterbalanced by shorter growing seasons. Increased production may result in increased carrying capacity on Bay Area rangelands. However, shorter growing seasons and increased potential for drought will increase risk. One of the primary tools for reducing drought risk is to maintain stocking rate below the carrying capacity of the land, which means the 10 to 25 percent increases in forage production forecast here may not result in substantial increases in stocking rate. Because drought is a regular occurrence on Bay Area rangelands, especially the south eastern portion that lies in the rain shadow of the Coast Range, ranchers in these areas are already accustomed to coping with periodic drought. For the climate scenarios discussed above, grazing managers will need to strengthen their contingency planning for drought.

Overall, this model has demonstrated that shifting temperature and precipitation patterns must be considered together in order to understand the potential impacts of climate change on rangeland forage production. In a future with higher temperatures and a shorter rainy season, ranchers will need to consider management options for grazing shorter growing seasons and therefore longer dry seasons. Most of the standing biomass remaining during the dry season has senesced and is of poor nutritive quality; an extension of this period means a reduction in the availability of forage that can meet the nutrition requirements of beef cattle. These vulnerabilities to climate change are not as easily translated to economic impacts as total forage production, as each ranch has a unique set of forage sources and operational conditions. Some ranches have the flexibility to transport livestock to forage sources of higher quality during the Bay Area dry season (e.g., irrigated pastures, high elevation meadows, or wetter coastal regions), while others will graze the dry forage remaining in the Bay Area and therefore need to provide supplemental feeds including hay, protein and mineral supplements. Both of these options will increase production costs, reducing already thin profit margins, but how these additional costs will weigh against the projected gains in forage production is not well understood. However, the main message for the effects of climate change on Bay Area ranching is that it will present some opportunities as well as some challenges. The prospect of paying ranchers to graze in order to provide certain ecosystem services such as control of invasive species [27], fire hazard reduction [28], and pollination to nearby farms [29] may be an increasingly important tool to help offset the increased costs of grazing under climate change and to maintain the viability of ranching operations in the Bay Area – and the precious open spaces they support.
